# A systematic review and meta‐analysis of the prognostic role of age in oral tongue cancer

**DOI:** 10.1002/cam4.3795

**Published:** 2021-03-24

**Authors:** Marta Tagliabue, Pietro Belloni, Rita De Berardinis, Sara Gandini, Francesco Chu, Stefano Zorzi, Caterina Fumagalli, Luigi Santoro, Susanna Chiocca, Mohssen Ansarin

**Affiliations:** ^1^ Division of Otolaryngology and Head and Neck Surgery IEO European Institute of Oncology IRCCS Milan Italy; ^2^ Department of Experimental Oncology IEO European Institute of Oncology IRCCS Milan Italy; ^3^ Department of Statistical Sciences University of Padua Padua Italy; ^4^ Division of Pathology IEO European Institute of Oncology IRCCS Milan Italy; ^5^ Chiesi Farmaceutici Parma Italy

**Keywords:** age, meta‐analysis, oral cavity cancer, survival, tongue cancer, young adults

## Abstract

While evidence suggests an increasing incidence of tongue cancer in young adults, published findings regarding the prognostic role of age at diagnosis are inconsistent. We performed a meta‐analysis of the literature to highlight key points that might help in understanding the association between age of oral tongue cancer patients at diagnosis and their prognosis. According to age at diagnosis, a systematic literature review of all published cohort studies assessing the recurrence risks and mortality associated with tongue cancer was conducted. We compared the risk estimates between patients aged >45 years and those aged <45 years at diagnosis. Random‐effects models were used to calculate summary relative risk estimates (SRRs) according to different clinical outcomes and sources of between‐study heterogeneity (I^2^) and bias. We included 31 independent cohort studies published between 1989 and 2019; these studies included a total of 28,288 patients. When risk estimations were not adjusted for confounders, no significant association was found between age at diagnosis and overall survival (OS). Conversely, after adjustment for confounders, older age at diagnosis was associated with a significantly increased risk of mortality. The difference between SRRs for adjusted and unadjusted estimates was significant (*p* < 0.01). Younger patients had a significantly higher risk of local recurrence. Younger patients with oral tongue cancer have better OS but a greater risk of recurrence than older patients. These findings should be validated in a large prospective cohort study which considers all confounders and prognostic factors.

## Graphical abstract

Although oral tongue cancer is generally considered to affect older patients, increasing evidence suggests an increasing incidence in younger patients. The study results illustrated that age at diagnosis had varying effects on the risks of poor outcomes and local recurrence. We suggest that younger patients with oral tongue cancer should receive personalized follow‐up plans to improve prognosis by identifying disease recurrence at an early stage.

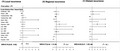

## 1 INTRODUCTION

Oral cavity cancer (OCC) is the most common malignancy of the head and neck region, as reported by the Global Cancer Observatory (GLOBOCAN).^1^ The incidence of new OCC is expected to exceed 29/100,000 persons worldwide by 2030 in both men and women at all ages (http://gco.iarc.fr/tomorrow).^2^

In light of these epidemiological data, Authors show an increasing interest about OCC and oral tongue squamous cell cancer (OTSCC), because of the crucial role of this anatomical region and the impact of therapies and treatments on swallowing and respiration.^2^, ^3^, ^4^, ^5^, ^6^ More recently, the authors have voiced concern about an increasing trend of the incidence of OSTCC among the “young population,” these data are reported for Scotland, the US, and Northern Europe in addition to India, China, and South Korea, although an unequivocal definition of this subgroup of patients has not been released yet and globally accepted.^3^, ^7^, ^8^, ^9^, ^10^, ^11^, ^12^ In the “young population,” the female group under the age of 45 seems more affected, with gender differences between female and male patients regarding the age at diagnosis and tumor site^3^, ^12^, ^13^ Focusing in the whole female group, older women (>70 years) seem to be more affected than younger women, suggesting a cumulative etiological effect leading to cancer. Male patients with an age of 51 to 60 years are mostly affected by buccal mucosa carcinoma, while female patients mainly develop oral tongue tumors. However, there are no differences in OS between male and female patients, suggesting that patients with OSCC have similar survival conditions.^13^

Typically, alcohol consumption, cigarette smoking, and betel nut chewing are prominently males' habits. This could be one explanation for the earlier age at diagnosis of OSCC in male patients, compared with females.^14^, ^15^

Tongue cancer has always been considered to affect primarily middle‐aged men, and it has been associated with tobacco and alcohol use.^10^, ^16^ In Southeast Asia, OSCC is associated with chewing betel nut, a traditional habit.^10^, ^16^ Nevertheless, compared with older patients, young patients have no history of tobacco or alcohol abuse. Furthermore, the time of exposure to these well‐known risk factors may not have been sufficient to promote malignant transformation, suggesting the involvement of other external or internal factors in the development of tongue cancer in this population.^17^, ^18^, ^19^, ^20^, ^21^ In the past years, an increasing incidence of TSCC has been registered in those countries where the primary prevention campaign to stop smoking and drinking has been active, thus suggesting the possible emerging role of new etiological or genetic factors driving carcinogenesis.^5^, ^10^, ^11^, ^22^, ^23^, ^24^

More recently, the authors have investigated other independent risk factors for OSCC in young patients as chronic mucosal trauma, poor oral hygiene, or inadequate dental status.^25^, ^26^

By studying the genomic profiles, similar mechanisms of tumorigenesis were discovered to be very similar among young and elderly OTSCC patients.^27^, ^28^, ^29^

Nowadays, the role of human papillomavirus (HPV) for oral tongue cancer appears undefined, but yet different from the oropharynx.^30^, ^31^ Dietary nutrients, specifically fruits and vegetables, have been consistently associated with lower oral cancer risk.^32^

In summary, exposure to tobacco, excessive alcohol consumption, and a diet with low intake of fruits and vegetables combined with poor oral hygiene, significantly increase the risk of oral cancer in the whole population.

The women subgroup OTSCC is not often associated with HPV infection, tobacco, and alcohol consumption. Since this disease does not appear to be related to dietary habits, it may be considered a new emerging and distinct clinical entity.^13^, ^23^

In young adults, the role of prognostic factors such as genetic or histopathological alterations has been studied.^33^, ^34^, ^35^ In this regard, the discussion has focused on the role of unknown risk and prognostic factors in young patients with OTSCC. Several authors have reported that compared with older patients, younger patients have worse outcomes in overall survival (OS), disease‐free survival (DFS), or recurrence rate,^17^, ^18^, ^19^, ^36^, ^37^ with women having a higher risk of poor outcomes than men.^19^, ^23^ For instance, Park et al. revealed that patients aged <45 years at diagnosis of advanced‐stage oral cancer have a worse overall regional recurrence and DFS rates than their older counterparts.^38^ Conversely, other authors have reported that younger patients have similar or better prognoses than older patients.^39^, ^40^, ^41^, ^42^, ^43^, ^44^, ^45^, ^46^, ^47^, ^48^ Therefore, the predictive value of age must be studied to optimize the treatment of OTSCC in young patients.

In the present study, we performed a comprehensive meta‐analysis of all published data regarding the prognosis of younger patients (≤45 years) versus older patients (>45 years) to better understand the role of age at diagnosis in the prognosis of OTSCC.

## 2 METHODS

We performed a systematic literature review and searched for studies that assessed the impact of age at diagnosis on the prognosis of patients with OTSCC. We conducted a meta‐analysis to evaluate the association of age at diagnosis with DFS (local, regional, or distant recurrence) and OS. This study was conducted according to the Meta‐analysis of Observational Studies in Epidemiology guidelines.

### 2.1 Information sources and search strategy

Scientific literature published up to June 2019 was searched using the Medline, Embase, and Web of Science databases by three independent reviewers (MT, LS, and RDB) to identify papers, and relevant data were extracted. Disagreements among reviewers were resolved by discussion. The search strategy was consistent across the databases, and it was performed using the following keywords: “Tongue Neoplasms” [MeSH Major Topic] AND (young OR younger OR older OR elderly OR age). To ensure that all studies assessing any outcome of interest were captured, no selective keywords referring to the outcome were introduced in the search strategy. Cross‐referencing from relevant studies was performed to confirm the retrieval of all possible studies. There were no restrictions in the search in terms of the year of publication or language.

### 2.2 Selection of articles

The inclusion criteria for this meta‐analysis were as follows:

▪ Studies that investigated the prognosis of oral tongue cancer, with the anatomical sites being the anterior two‐thirds, dorsal surface, tip, and lateral border of the tongue.
▪ Studies must be independent to avoid giving double weight to estimates derived from the same study.
▪ Studies must present risk estimates or data to extract risk estimates by age.
▪ Studies must include data on at least one of the following clinical outcomes: local recurrence‐free survival (LRFS), regional recurrence‐free survival (RRFS), distant recurrence‐free survival (DRFS), DFS, disease‐specific survival (DSS), or OS.
▪ Studies must present adjusted hazard ratios (HRs) and 95% confidence intervals (CIs) comparing outcomes among age groups: younger patients (≤45 years) versus older patients (>45 years); if adjusted HRs were not presented, then eligible studies had to provide sufficient information to estimate either the HR or the relative risk (RR) and the corresponding standard errors and 95% CIs.
▪ Studies wherein all patients were treated according to their tumor stage and in accordance with internationally recognized guidelines.^49^

### 2.3 Exclusion criteria

The exclusion criteria for this meta‐analysis were as follows:

▪ Studies with an unspecified definition of “tongue cancer,” with no distinction between the base of the tongue and mobile tongue
▪ Studies examining the base of the tongue because cancer at this location is formally considered oropharyngeal tongue cancer

### 2.4 Statistical analysis

All association and corresponding 95% CIs were log‐transformed, and the corresponding variance and standard error were calculated using the formula proposed by Greenland.^50^ When estimates were not available from the paper, we calculated them from the published crude data in terms of RRs. Woolf's formula was used to obtain the standard error of log RR. Finally, if only survival curves were presented, then HR and its log‐transformed value and standard error were indirectly extracted using Parmar's method.^51^

The association of age group with outcomes across studies was computed as summary RRs (SRRs) with 95% CIs by pooling the study‐specific estimates using a random‐effects model fitted using the R statistical software (version 3.6.0). These models provided estimates adjusted for the potential correlation within studies as well as the heterogeneity among studies.

The homogeneity of the effect across studies for large samples was assessed using Cochrane's Q test, which is approximately distributed as χ^2^ statistic, for which *p* < 0.10 was used to indicate the lack of homogeneity among effects. I^2^ statistic was also calculated to quantify the percentage of total variation across studies attributable to the study's heterogeneity rather than to the chance.^52^ The methods reported by Sterne et al. was used to assess publication bias.^53^

Sensitivity, subgroup, and meta‐regression analyses were conducted to investigate heterogeneity among studies focusing on the study characteristics, such as country, the cutoff for age, main tumor stage of included patients, year of publication, and adjustment for confounders and other prognostic factors.

## 3 RESULTS

### 3.1 Description of the retrieved studies

A total of 3678 studies were retrieved using the aforementioned keywords, and of these, 279 were related to OTSCC, young age, and prognosis and were considered for further scrutiny. We excluded studies that did not meet the previously defined inclusion criteria.

**FIGURE 1:**
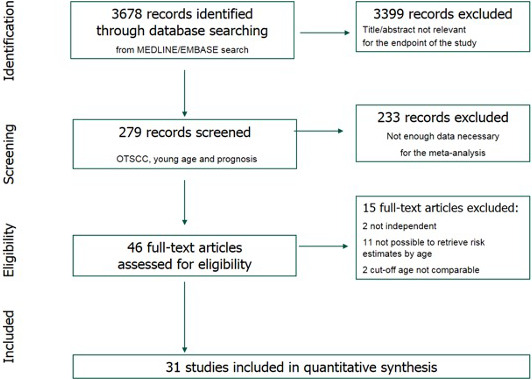
PRISMA flow chart.

In particular, we filtered out studies that did not specifically address mobile oral cancer, studies that had a cutoff age at diagnosis of OTSCC other than 40 or 45 years, review articles, case reports, and editorials. After applying these inclusion/exclusion criteria, 44 studies were considered potentially eligible. Two additional studies that used an age cutoff of 30 years were forced to be included because of the reported data's strong validity. We then excluded studies with insufficient statistical information to estimate the risk of recurrence or mortality according to age (15 full‐text articles were excluded). Details of the excluded studies are summarized in PRISMA flowchart (Figure 1).

Finally, we included 31 independent studies published between 1989 and 2019; these studies included a total of 28,288 patients (Table 1). Of the 31 studies, six were prospective, and the remaining were retrospective. The studies were conducted in different regions as follows: 5, 9, 9, 3, 2, 1, 1, and 1 in Europe, the USA, Asia, Israel, Australia, Saudi Arabia, Canada, and Brazil, respectively. The largest study was also the most recent one, four conducted in 22,930 patients in the USA. Some studies presented risk estimates adjusted for confounders or other prognostic factors [sex, tumor–node–metastasis (TNM) stage, smoking status, alcohol consumption, ethnicity, adjuvant therapy, lymphatic and vascular invasion, surgical treatment, comorbidities, and surgical margins].

**TABLE 1:** Descriptive characteristics of the included cohort studies

First author, PY	Study design	Country	Cutoff age (years)	No. of patients	T stage	N stage	Outcomes
Jones, 1989^54^	NA	Canada	40	71	NA	NA	OS
Siegelman, 1998^48^	R	USA	45	87	Any	Any	OS, DFS
Friedlander, 1998^42^	R	USA	40	72	Any	Any	DFS
Yoshida, 1999^55^	NA	Japan	40	568	T1, T2	N0	OS, DSS
Al Rajhi, 2000^56^	NA	Saudi Arabia	45	85	T1, T2	N0	OS, DSS
Vargas, 2000a^46^	R	USA	40	34	Any	Any	OS, DFS
Pitman, 2000^43^	R	USA	40	272	Any	Any	DFS
Davidson, 2001^57^	P	USA	40	819	NA	NA	OS, DSS
Veness, 2003^47^	R	Australia	40	164	Any	Any	OS, DFS
Hyam, 2003^36^	R	Australia	40	144	Any	Any	OS
Popovtzer, 2004^39^	P/R	Israel	45	48	T1, T2	Any	OS, DSS
Liao, 2006^45^	P	Korea	40	296	Any	Any	OS, DSS, DFS
Garavello, 2007^17^	P	Italy	40	138	Any	Any	OS, DSS, DFS
Lee, 2007^58^	R	Taiwan	45	40	Any	Any	OS, DSS
Lim, 2007^59^	R	Korea	45	32	T2	N0	OS, DSS
Yang, 2008^60^	P	China	40	229	Any	Any	OS
Park, 2010^38^	R	Korea	40	85	Any	Any	OS, DSS
Soudry, 2010^44^	R	Israel	30	85	Any	Any	OS, DSS, DFS
Kies, 2012^61^	R	USA	40	23	T2, T3	N0–N2	OS, DSS
Kabeya, 2012^62^	R	Japan	40	32	Any	Any	OS, DFS
Hilly, 2013^18^	R	Israel	30	78	Any	Any	OS, DSS, DFS
Chen, 2016^63^	P	Taiwan	40	128	Any	Any	DSS
Sgaramella, 2015^64^	NA	Italy, Sweden	40	129	Any	Any	OS
Santana, 2017^21^	R	Brazil	45	82	Any	Any	DSS, DFS
Mroueh, 2017^41^	R	Finland	40	563	Any	Any	OS, DSS
Cassidy, 2017^65^	R	USA	45	180	Any	N0	OS
Blanchard, 2017^40^	R	France	40	100	Any	Any	OS, DSS
Knopf, 2015^66^	R	Germany	45	276	Any	Any	OS, DSS
Chen, 2018^67^	R	China	45	101	Any	Any	OS
Farquhar, 2018^68^	R	USA	45	397	Any	Any	OS, DSS
Oliver, 2019^4^	R	USA	40	22,930	Any	Any	OS

PY, Publication year; P, prospective; R, retrospective; NA, not available; DFS, disease‐free survival; DSS, disease‐specific survival; OS, overall survival. DFS may also include local recurrence‐free survival, regional recurrence‐free survival, and distant recurrences‐free survival.

^a^ Study included only women.

### 3.2 Meta‐analysis results

**FIGURE 2:**
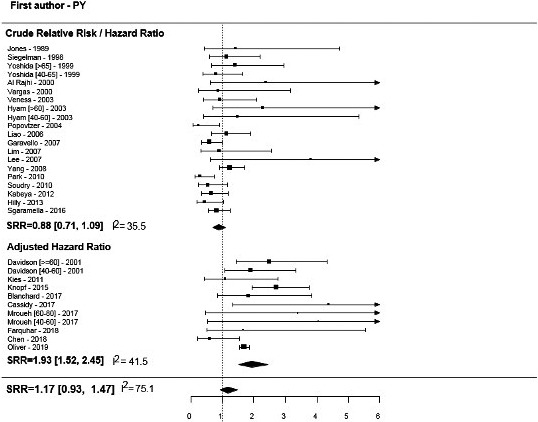
Overall survival for older versus younger OTSCC patients.

**FIGURE 3:**
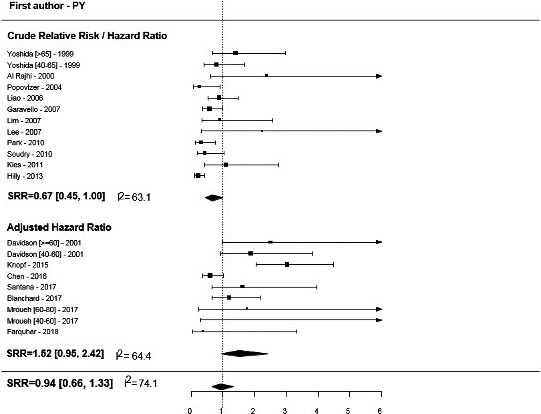
Disease‐specific free survival for older versus younger OTSCC patients.

Overall, we found no association of age with OS and DSS, but pooled estimates were significantly different between crude and adjusted HRs (forest plots in Figures 2 and 3; *p* < 0.01, Table 2). Summary HR estimates from multivariate models adjusted for confounders indicated a nearly twofold increase in the risk of mortality in older patients versus younger patients (SRR = 1.93, 95% CI = 1.52–2.45), with no significant between‐study heterogeneity (I^2^ = 41.5%). Although statistically insignificant, the inverse association was found for unadjusted risk estimates, with older age being associated with an approximately 10% reduction in the risk of mortality (SRR = 0.88, 95% CI = 0.71–1.09, I^2^ = 35.5%), and a similar trend was found for DFS.

**TABLE 2:** Summary relative risk for older vs. younger patients from random‐effects models by tumor outcome and after adjustment for confounders of the risk estimates

		No. of estimates	SRR	Lower 95% CI	Upper 95% CI	*p*‐value	I^2^ (%)
OS	Overall	31	1.17	0.93	1.47		76
	Crude	20	0.88	0.71	1.09	<0.01	35
	Adjusted	11	1.93	1.52	2.45		41
DSS	Overall	21	0.94	0.66	1.33		74
	Crude	12	0.67	0.45	1.00	0.01	63
	Adjusted	9	1.52	0.95	2.42		64
DFS	Crude	11	0.76	0.6	0.95		31
LRFS	Crude	12	0.76	0.63	0.92		0
RRFS	Crude	12	0.75	0.48	1.17		73
DRFS	Crude	12	0.60	0.27	1.35		66

*P*‐value from meta‐regression

SRR, summary risk estimate; CI, confidence interval; OS, overall survival; DSS, disease‐specific survival; DFS, disease‐free survival; LRFS, local recurrence‐free survival; RRFS, regional recurrence‐free survival; DRFS, distant recurrence‐free survival

**FIGURE 4:**
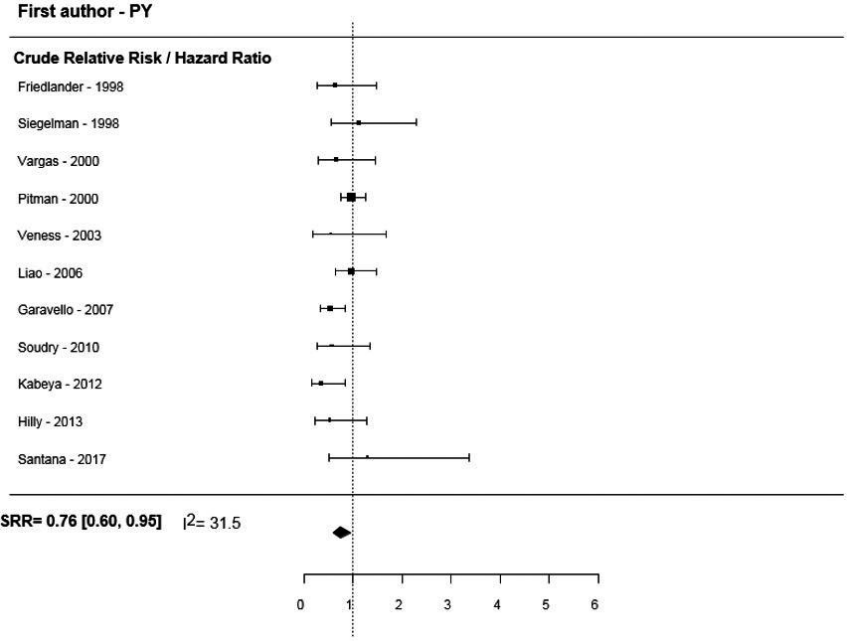
Disease‐free survival for older versus younger OTSCC patients.

**FIGURE 5:**
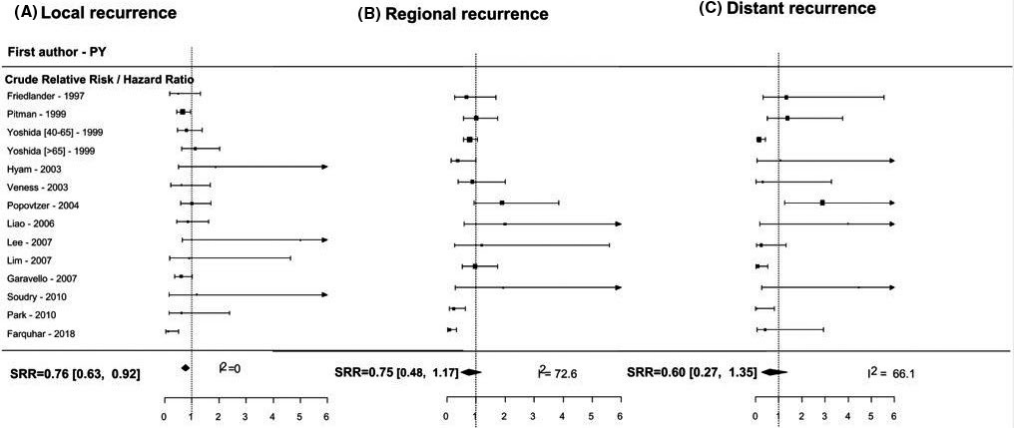
(A) Local recurrence for older versus younger OTSCC patients. (B) Regional recurrence for older versus younger OTSCC patients. (C) Distant recurrence for older versus younger OTSCC patients.

Overall, 11 studies presented unadjusted estimates for DFS (forest plot in Figure 4), and the SRR estimate indicated a 24% reduction in the risk of local recurrence in older patients, with no between‐study heterogeneity (SRR = 0.76, 95% CI = 0.63–0.92, I^2^ = 0). Similar results were found for the summary unadjusted RR/HR estimates of DFS with low heterogeneity (I^2^ = 31%). In contrast, the summary estimates for DRFS and RRFS, despite displaying similar patterns in terms of the point estimate, were found to be statistically insignificant (Table 2 and Figure 5A,B,C).

Regarding sensitivity analyses, exclusion of the most recent and largest cohort study, which accounted for 80% of the total patient cohort included in the OS analyses, did not affect the results (SRR = 2.02, 95% CI = 1.48–2.74).^4^ The majority of the studies compared outcomes by age using 40 or 45 years as the cutoff,^4^, ^17^, ^54^, ^55^, ^56^, ^57^, ^58^, ^59^, ^60^, ^61^, ^62^, ^63^, ^64^, ^65^, ^66^, ^67^, ^68^ except for studies by Hilly et al. and Soudry et al. that used 30 years old as the cutoff.^18^, ^44^ Both these studies presented non‐adjusted risk measures and indicated that older patients had lower risks of recurrence and mortality than their younger counterparts. The sensitivity analysis of the crude summary estimate for OS confirmed that no association existed after excluding these two studies (SRR =0.94, 95% CI = 0.76–1.16). Besides, the summary estimates for local recurrence did not change (data not shown).

Hyam et al., Yoshida et al., and Davidson et al. presented estimates for two cutoff points: “very old” (>60–65 years) versus “younger” (<40–45 years) and “middle age” (between 40–50 and 60–65 years) versus “younger” (<40–45 years).^36^, ^55^, ^57^ These studies, except for Mroueh et al.,^41^ recorded a higher risk of recurrence of mortality for very old patients versus younger patients and very old patients versus middle‐aged patients (forest plots in Figures 2 and 3).

Other sources of between‐study heterogeneity were investigated using subgroup analyses and meta‐regression (Table 3). We found a significant difference (*p* = 0.03) in risk estimates by country, such that older patients had a significantly higher risk of mortality in the USA or Australia. In contrast, the inverse association was observed in East Asian and Middle Eastern countries, albeit without significance in some cases. We found a significantly reduced risk of local recurrence for the same countries, but none of the estimates was adjusted for other prognostic factors.

**TABLE 3:** Subgroup analyses and results of meta‐regression models

Outcome			No. of studies	SRR	Lower 95% CI	Upper 95% CI	*p*‐value
OS	pT	T1–T2 only	6	0.97	0.87	1.45	0.60
		Any pT	22	1.13	0.85	1.49	
	pN	N0 only	5	1.32	0.85	2.06	0.37
		Any pN	23	1.03	0.78	1.37	
	Region	Europe	6	1.45	0.77	2.75	0.03
		USA/Australia	12	1.69	1.55	1.84	
		East Asia	9	0.88	0.65	1.20	
		Middle East	4	0.57	0.75	1.17	
DSS	pT	T1–T2 only	6	0.97	0.65	1.45	0.69
		Any pT	13	0.81	0.50	1.18	
	pN	N0 only	4	1.12	0.72	1.74	0.31
		Any pN	15	0.75	0.47	1.21	
	Region	Europe	5	1.41	0.67	2.96	0.09
		USA/Australia	5	1.64	1.08	2.48	
		East Asia	7	0.78	0.55	1.09	
		Middle East	4	0.44	0.46	1.18	
DFS	pT	T1–T2 only	0	‐	‐	‐	
		Any pT	11	0.76	0.60	0.95	‐
	pN	N0 only	0	‐	‐	‐	
		Any pN	11	0.76	0.60	0.95	‐
	Region	Europe	1	0.53	0.35	0.84	0.14
		USA/Australia	6	0.93	0.75	1.15	
		East Asia	2	0.64	0.25	1.65	
		Middle East	2	0.56	0.31	1.02	
Local RFS	pT	T1–T2 only	4	0.96	0.70	1.31	0.06
		Any pT	10	0.67	0.52	0.84	
	pN	N0 only	3	0.94	0.64	1.38	0.21
		Any pN	11	0.71	0.57	0.88	
	Region	Europe	1	0.61	0.37	1.02	0.15
		USA/Australia	5	0.58	0.34	0.98	
		East Asia	6	0.93	0.68	1.28	
		Middle East	2	1.01	0.61	1.69	
Regional RFS	pT	T1–T2 only	2	0.79	0.58	1.06	0.74
		Any pT	10	0.72	0.41	1.25	
	pN	N0 only	2	0.79	0.58	1.06	0.74
		Any pN	10	0.72	0.41	1.25	
	Region	Europe	1	0.96	0.53	1.75	0.57
		USA/Australia	5	0.51	0.24	1.08	
		East Asia	5	0.94	0.44	2.01	
		Middle East	1	1.93	0.28	13.35	
Distant RFS	pT	T1–T2 only	1	0.16	0.06	0.42	0.21
		Any pT	11	0.73	0.32	1.67	
	pN	N0 only	1	0.16	0.06	0.42	0.21
		Any pN	11	0.73	0.32	1.67	
	Region	Europe	1	0.25	0.05	1.31	0.13
		USA/Australia	5	1.01	0.50	2.04	
		East Asia	4	1.33	0.23	7.78	
		Middle East	2	0.08	0.02	0.35	

*P*‐value from meta‐regression for differences between subgroups

SRR, summary risk estimate; CI, confidence interval; OS, overall survival; DSS, disease‐specific survival; DFS, disease‐free survival; RFS, recurrence‐free survival; pT, pathological tumor; pN, pathological node.

SRR was estimated for older patients vs. younger patients.

No publication bias, as measured by the method of Sterne et al.^53^ was observed for any outcome (crude OS, *p* = 0.51; adjusted OS, *p* = 0.90; DSFF, *p* = 0.55; DFS, *p* = 0.19; LRFS, *p* = 0.61; RRFS, *p* = 0.78; and DRFS, *p* = 0.87).

## 4 DISCUSSION

The present meta‐analysis aimed to reveal the difference in prognosis between younger and older patients with OTSCC.

In the vast majority of published studies, the “young group” is not uniformly defined, or its definition is based on an arbitrary judgment, with cutoffs ranging from 20 to 30, 40, or 45 years. In addition, differences in the median/mean age among age groups ranged from a minimum of 12 years to a maximum of 42 years.^44^, ^58^ Among the 31 selected studies, the “younger patients” group, in terms of age at diagnosis, was defined as <30, <40, and <45 years in 3, 15, and 9 studies, respectively, whereas “older patients” was defined with consecutive ranges in the remaining studies. We used the 45‐year cutoff for the current analysis to include all the studies mentioned above with age ranges of 30, 40, and 45 years, to maximize the possible study population, and consider that the definition of "young adult" is still not universal defined.

The increasing interest for “young patients” affected by oral cancer, is related to the different etiological factors which might differentiate this subset of patients, and indirectly influence their prognosis, response to treatments and prompt recovery compared to aged patients. Prognosis and function rehabilitation is closely related to the quality of life in all patients affected by oral cancer. Nevertheless, this is even more crucial for younger patients since the sequelae of OTSCC treatments and surgery impair patients’ future personal and professional perspectives and social skills. Nowadays, researchers’ main challenge is to identify an ideal treatment that might optimize the cost‐benefit ratio and provide good prognosis with minimal functional impairment. Moreover, besides smoke and alcohol consumption, identifying new risk factors could lead to the establishment of prevention measures against tongue cancer especially in young patients and redefine patients’ risk stratification for tailored surveillance and follow‐up programs.^19^

Although the “infectious aspect” of tumors in younger patients is currently being investigated, the relationship between HPV infection and tongue cancer risk in young patients remains debatable. Compared with that observed for oropharyngeal cancer, viral infection is not strongly related to tongue cancer.^69^, ^70^, ^71^ However, abnormal dentition in terms of persistent trauma and chronic inflammation and a diet low in fruit and vegetables have been studied at length, providing consistent results.^25^, ^32^, ^72^ Finally, the role of genetic alterations such as an altered expression (overexpression) of the epidermal growth factor receptor in young adults has been reported to be related to a worse prognosis.^34^, ^35^

The literature is very heterogeneous with regards to the prognosis by age group, ranging from a worse prognosis for young people to a significantly better prognosis.^4^, ^36^

In the current analysis, younger patients exhibited better prognosis in terms of OS but a higher risk of recurrence, even if the estimations were not adjusted for confounders and other prognostic factors. Conversely, older patients exhibited a lower risk of local recurrence and worse DSS according to estimates that were not adjusted for other prognostic factors and possible confounding factors such as TNM stage, sex, and treatment type. This trend was also confirmed in other studies which presented two different cutoffs for age: Mroueh et al. and Davidson et al. reported that older patients (>60 years at diagnosis) had worse prognoses than patients in the reference group (40–60 years at diagnosis).^41^, ^57^

The findings of a higher risk of local recurrence and improved OS in young adults could be related to the assumption that younger patients benefit from a higher number of available therapeutic options, such as major surgery and first‐line chemotherapy. Generally, younger patients have fewer comorbidities and better resilience. Therefore, they may have lower probabilities of postoperative complications, thereby increasing their survival chance.^73^ Moreover, the increased recurrence rates may be associated with non‐cancer‐related mortality in older patients before relapse occurs.^73^

Furthermore, an interesting aspect of this study was represented by the heterogeneity of the survival outcomes concerning geographical areas: older patients seemed to have a significantly higher risk of mortality in the USA or Australia, whereas an inverse association was observed in East Asian and Middle Eastern countries. These differences can be justified by differences in health care systems among these countries and relative ease to access to medical care. In Asian, Middle Eastern countries as well as Latin America, limited access to medical care could be the cause of a delayed diagnosis and worse prognosis in younger people compared to the elderly when considering both OS and DSS.^74^ Countries with limited access to cure suffer from late diagnosis, treatments might be inadequate, with higher mortality and risk of relapse due to advanced‐stage disease.

The authors debate on the geographical prognostic differences between the elderly and young people also mirrored the incidence of disease: it seems that in Asia, Africa, and the Middle East, tongue cancer incidence among young population is highest. One explanation could be the lower life expectancy in these countries or an overestimated number of young patients because of the reluctance of elderly to attend a hospital.^3^

Our study has some limitations: it is not easy to clarify these aspects using published retrospective studies because the reported incidence of OTSCC in young adults is at <5%.^40^, ^75^ Moreover, different studies have presented discordant data, making it difficult to identify and establish the real impact of age on tongue cancer prognosis.^7^ The disparity of these results could be attributed to the small sample size of younger patients as well as the heterogeneity among studies (matched/unmatched studies, early/advanced tumor stage, different treatments reported, and cancer at different subsites [i.e., oral tongue cancer or cancer of the base of the tongue] considered in the same patient group).^69^, ^76^ The disparity could also be partly associated with the different definitions of “young” patients and different inclusion criteria regarding the anatomical tumor site. In many studies, the base of the tongue was included, although it is considered part of the oropharynx, which differs from tongue cancer for staging and prognosis.^76^, ^77^ Furthermore, the analyzed studies were conducted over an extremely long period during which the knowledge, diagnostics, and therapies in these fields have considerably changed. The majority of the included patients were part of a single recently published study in the USA. However, the effects of confounding factors were statistically lower.^4^

## 5 CONCLUSION

This systematic review and meta‐analysis revealed that younger patients with OTSCC have a better OS but a higher risk of recurrence than elderly patients. Therefore, they should receive personalized follow‐up plans to improve their prognosis, by identifying disease recurrence at an early stage and ensuring adequate oncological radicality with as much as possible functional conservative treatment.

Further prospective international studies with uniform inclusion criteria are strongly needed to corroborate our findings. Prospective studies could focus on different trends in women and teenagers, where the hormonal, genetic and molecular factors are potentially crucial.

## CONFLICT OF INTEREST

Nothing to declare.

## ETHICAL STATEMENT

Ethical approval is not be required because this study retrieves and synthesises data from already published studies.

## Data Availability

Data are available upon request.
